# Treatment of cancer cells with chemotherapeutic drugs results in profound changes in expression of genes encoding aldehyde-metabolizing enzymes

**DOI:** 10.7150/jca.32608

**Published:** 2019-07-10

**Authors:** Olga L. Zinovieva, Evgeniya N. Grineva, George S. Krasnov, Dmitry S. Karpov, Andrei O. Zheltukhin, Anastasiya V. Snezhkina, Anna V. Kudryavtseva, Tamara D. Mashkova, Nikolai A. Lisitsyn

**Affiliations:** 1 Engelhardt Institute of Molecular Biology, the Russian Academy of Sciences, 119991 Moscow, Russia; 2 Institute of Biomedical Chemistry, the Russian Academy of Sciences, 119121 Moscow, Russia

**Keywords:** colorectal cancer, chemotherapeutic drugs, reactive oxygen species, RNA-seq, serum biomarkers.

## Abstract

Using RNA-seq, RT-qPCR, and bioinformatics we have studied the influence of a wide spectrum of chemotherapeutic drugs on transcription of *AKR1B10*, *AKR1C1*, *ALDH1A1*, and *ALDH1A3* genes, which encode the major aldehyde-metabolizing enzymes. The strongest alterations were detected in case of AKR1B10 mRNA that was significantly upregulated in wild type p53 cancer cells, but downregulated in mutant p53 cancer cells. Subsequent experiments demonstrated the significant and consistent decrease in the AKR1B10 mRNA content in sera of colon cancer patients, as compared to sera of healthy donors (p<0.0001, SPE=92.9%, SNE=79.3%, AUC=0.889), which implies that this RNA is a valuable marker for serological diagnosis of colorectal cancer. Moreover, we have found that ALDH1A3 protein is a key inactivator of ROS-generated aldehydes, which is a perspective target for the development of new chemotherapeutic drugs.

## Introduction

Numerous data indicate that failure of cancer patients' chemotherapy is a result of drug resistance, which is acquired by cancer cells in course of the treatment. It arises as a result of transcriptional inactivation of three groups of genes, which encode: 1) drug-detoxifying enzymes; 2) inhibitors of apoptosis; and 3) drug transporters from the multi-drug resistance family 1. Enzymes encoded by the first group of genes catalyze inactivation of toxic aldehydes and other carbonyls, which are generated upon drug treatment as a consequence of the hyperproduction of the reactive oxygen species (ROS) and their interaction with membrane lipids, proteins, and DNA. ROS are constantly generated in normal cells and inactivation of their toxic byproducts is tightly controlled by maintenance of the optimal expression levels of numerous defensive antioxidative proteins. Treatment of cancer cells with most of the chemotherapeutic drugs results in ROS hyperproduction and cellular oxidative stress, which is a consequence of an imbalance between the production of toxic compounds and the ability of the cell to provide their detoxification. Failure to maintain the balance induces cell death through apoptosis, ferroptosis or necrosis.

The major targets of ROS-induced oxidative modifications are polyunsaturated lipids and lipoproteins of cellular membranes 2, 3, which are converted into hundreds of lipid peroxidation-derived aldehydes and ketones (LPO-derived carbonyls). Hyperproduction of ROS-generated carbonyls leads to transcriptional activation of genes encoding aldehyde-metabolizing enzymes, which belong to two major families of evolutionary conserved aldehyde scavengers: 1) aldehyde dehydrogenases (ALDHs) that catalyze the oxidation of aldehydes to carboxylic acids; and 2) aldo-keto reductases (AKRs), which reduce aldehydes to alcohols. It was shown that high expression levels of ALDHs and AKRs can protect cancer cells from the action of chemotherapeutic drugs 4, 5, as a consequence of either detoxification of ROS-generated toxic aldehydes or direct drug inactivation 6-8. Besides, AKRs increase survival rate of cancer cells by modulation of lipid biosynthesis and mitochondrial function 9.

Endogenous LPO-derived aldehydes (mainly HNEs and ONEs) are massively accumulated in cancer cells generating ROS upon drug treatment. The key regulators of the balance between ROS detoxification and ROS-generation are p53 and PI3K/AKT signaling pathways 10, 11, whereas the main effector of the antioxidant response is transcriptional factor NRF2. In normal conditions NRF2 is located in the cytoplasm in a complex with KEAP1 dimer and is constantly degraded by ubiquitination. Drug treatment of cells leads to accumulation of ROS-generated carbonyls in the cytoplasm, as a consequence of stimulation of NOX1 12 and enhanced leakage of byproducts of the oxidative phosphorylation from mitochondria 13. ROS hyperproduction results in the inactivation of KEAP1, dissociation of NRF2 and its translocation to the nucleus, where it forms a complex with sMAF. The NRF2-sMAF complex activates transcription of numerous genes, which contain antioxidant response elements (AREs) at their promoters (or up to 3 kb upstream), which results in rapid detoxification of ROS-generated carbonyls.

In this study we have analyzed the transcriptional response of the four genes encoding major aldehyde-metabolizing enzymes in colon cancer cell lines HCT-116 (p53wt) and HT-29 (homozygous for the gain of function mutation p53^R273H^). We have found the strongest differences in transcription of *AKR1B10* in p53wt and p53mut cancer cells, both *in vitro* and *in vivo* and demonstrated that this mRNA is a promising serological marker for colorectal cancer diagnosis.

## Materials and Methods

### Cell lines and drug treatment

Human colorectal cancer (CRC) cell lines HCT-116 and HT-29 were obtained from the American Type Culture Collection and grown for 72 h in DMEM/F12 medium (Invitrogen, USA) containing 10% fetal bovine serum, 4 mM L-glutamine, 100 units/mL penicillin, and 0.1 mg/mL streptomycin (Invitrogen, USA) at 37 °С and 5% СО_2_. HT-29 and HCT-116 cells were incubated for 72 h and 144 h in the medium that contained either 2 µM or 20 µM 5-FU, 2 µM or 20 µM OXP or 5 µM IRI (Sigma-Aldrich, USA). HT-29 cells were incubated for 72 h and 144 h in the medium that contained 2 µM or 20 µM 5-FU, 2 µM OXP or 5 µM IRI (Sigma-Aldrich, USA). HCT-116 cells were treated with OXP (2 µM or 20 µM OXP) for 72 h and 144 h.

### Collection of serum samples

Serum samples were collected from 43 subjects, including 29 patients with CRC and 14 healthy donors with no history of malignancies who served as the controls for this study. All samples were collected from consenting individuals in Herzen Moscow Oncology Research Institute - branch of National Medical Research Radiological Center, Ministry of Health of the Russian Federation. None of the patients received anticancer treatment prior to hospitalization. The tubes were centrifuged at 2,500 × g for 10 min at 4°C to completely remove cellular components. The serum was then collected gently and transferred into an RNase-free tube for the extraction of RNA and stored at -80°C. Prior to RNA extraction, stored supernatants were centrifuged again at 12,000 rpm at 4◦C for 15′ to remove cellular debris.

### RNA sequencing analysis

Total RNA samples were isolated from HT-29 cells untreated or treated with 20 µM 5-FU for 72 h, using the MagNA Pure Compact Instrument (Roche, Switzerland) according to the manufacturer's instructions (the procedure included DNase treatment). Purified RNA samples were quantified with Qubit 2.0 Fluorometer (Thermo Fisher Scientific, USA) and samples quality was estimated by the calculation of RNA Integrity Number (RIN), using Agilent 2100 Bioanalyzer (Agilent Technologies, USA). RNA samples with the RIN higher than 8.0 were used for subsequent analysis. Poly (A^+^) mRNA fraction was isolated from 1 μg of total RNA samples using NEBNext poly(A) mRNA Magnetic Isolation Module (New England Biolabs, USA). CDNA library preparation was carried out using NEBNext Ultra Directional RNA Library Prep Kit and NEBNext Multiplex Oligos for Illumina (New England Biolabs, USA) according to manufacturer's instructions. The quality and concentration of cDNA libraries were assessed as described above; cluster densities were optimized by qPCR, using Rotor-Gene Q 5 plex platform (Qiagen, Germany). Obtained cDNA libraries were sequenced in triplicate on the Illumina NextSeq 500 platform under the 2 × 43 bp paired-end model, yielding 170M mapped reads per experiment.

### Identification and ranking of differentially expressed transcripts

Illumina reads were trimmed by trimmomatic 14. Bacterial DNA/RNA contamination analysis of the obtained libraries was performed by mapping the sequencing reads to human rRNA and bacterial genomes databases using bowtie2 (100,000 reads were randomly selected for each sample). All the reports were summarized using MultiQC 15; trimmed reads were aligned to human genome GRCh38 (Ensemble annotation, release 88) using STAR aligner 16, and read counts per gene were estimated using HTSeq-count 17. All processing steps including quality control were performed using PPLine pipeline 18; the subsequent analysis was performed in R environment. Differential expression analysis was carried out using edgeR Bioconductor package (TMM normalization, likelihood ratio test) 19. In order to rank the genes we used the simple algorithm, which is based on calculation of gene expression scores according to the following formula:

S= (-log (*p*) · abs (log (*FC*) · (abs (*r*) + 0.2)^0.4^.

We have calculated the *p*-value using the edgeR's likelihood ratio (LR) test or quasi-likelihood (QL) F-test; *FC* is the expression level fold change; and *r* is the Spearman rank correlation coefficient. Analysis of transcripts from the public NCBI SRA RNA-Seq datasets was performed in the same way.

### Reverse transcription quantitative PCR

Total RNA samples were extracted from frozen tissues specimens and cultured colon cancer cells using the RNeasy Mini kit (Qiagen Inc., Germany). Serum RNA was isolated with mirVana PARIS kit (Ambion, USA). RNA integrity was evaluated by electrophoresis on 1% agarose gel and RNA quantity was determined using ND 1000 spectrophotometer (NanoDrop Technologies, Germany). First-strand cDNA was synthesized using 1µg of total RNA, random primers (Evrogen, Russia) and SuperScript^TM^ III reverse transcriptase (Invitrogen, USA). Obtained cDNAs were amplified in the presence of gene specific primers (Table S1), using the ABI 7500 Fast Real-Time PCR System (Applied Biosystems, USA); *ACTB* and *GAPDH* genes were used as controls. qPCR reactions were performed in triplicate in presence of the EvaGreen^TM^ dye (Biotium Inc., USA) in the following conditions: denaturation at 95 °C for 10 min followed by 40 cycles of amplification (95 °C for 15 sec, 60 °C for 1 min). Each plate included negative contamination control (in absence of cDNA); all experiments were repeated twice. Dissociation curve analysis was used in order to detect non-specific products. The relative expression ratios were calculated using the 2^-ΔΔCt^ method 20; fold change values (FC) ≥ 2 were considered to be statistically significant.

### Mouse tumor xenograft experiments

All animal experiments were performed in accordance with relevant ARRIVE guidelines and regulations. Ten Balb/c-nude mice (female, ages 4-6 weeks; Jackson Labs) were inoculated subcutaneously with 3 × 10^5^ HT-29 cells per mouse. When tumors reached a mean volume of 100 mm^3^, four mice were intraperitoneally injected with 10 mg/kg of 5-FU dissolved in 50 µL PBS (once a day for five days) and sacrificed three days after the completion of the treatment. IRI (40 mg/kg) was administered intravenously on the first and eighth day in six mice, which were euthanized with xylasine and sacrificed on the next day after completion of the treatment. Five control mice were intraperitoneally injected with buffered saline (once a day for five days) and sacrificed three days after the completion of the treatment. Total RNA samples were prepared from dissected tumors and serum and used for RT-qPCR as described above.

### Statistical analysis

ROC curves and related statistics were obtained using MedCalc Statistical Software version 16.8.4 (MedCalc Software bvba, Ostend, Belgium; 2016) with default parameters. Standard error was calculated according to 21.* P*-values < 0.05 were regarded as statistically significant.

## Results

### Transcription of the genes encoding aldehyde-metabolizing enzymes is significantly changed upon drug treatment of HT-29 and HCT-116 colon cancer cells

We have used RNA-Seq in order to identify changes in the transcriptome of HT-29 cells treated with 20 µM 5-FU for 72 h, as compared to untreated cells. We have used a high dose of the drug, which kills nearly 80% of cells 22, expecting that such treatment would selectively promote the survival of cells that acquired a transient resistance to 5-FU. RNA-Seq of reverse transcribed polyadenylated RNA samples obtained from the 5-FU treated and untreated HT-29 cells, allowed the robust measurement of the abundance of 14,580 genes of which 1,043 were upregulated and 1,991 downregulated (FDR < 0.01) with more than twofold changes in the expression level (logFC>1) (Table S2). Pathway analysis of the differentially expressed genes demonstrated a strong bias towards the transcripts encoding aldehyde-metabolizing enzymes including ten genes from the ALDH family and three genes from the AKR family. Surprisingly, transcription of all ten differentially expressed ALDH encoding genes was strongly downegulated in 5-FU treated HT-29 cells, as compared to control. The most notable change was detected for ALDH1A1 mRNA (13.8 times, the third on the top of the gene list with a maximum fold change).

**Figure 1:**
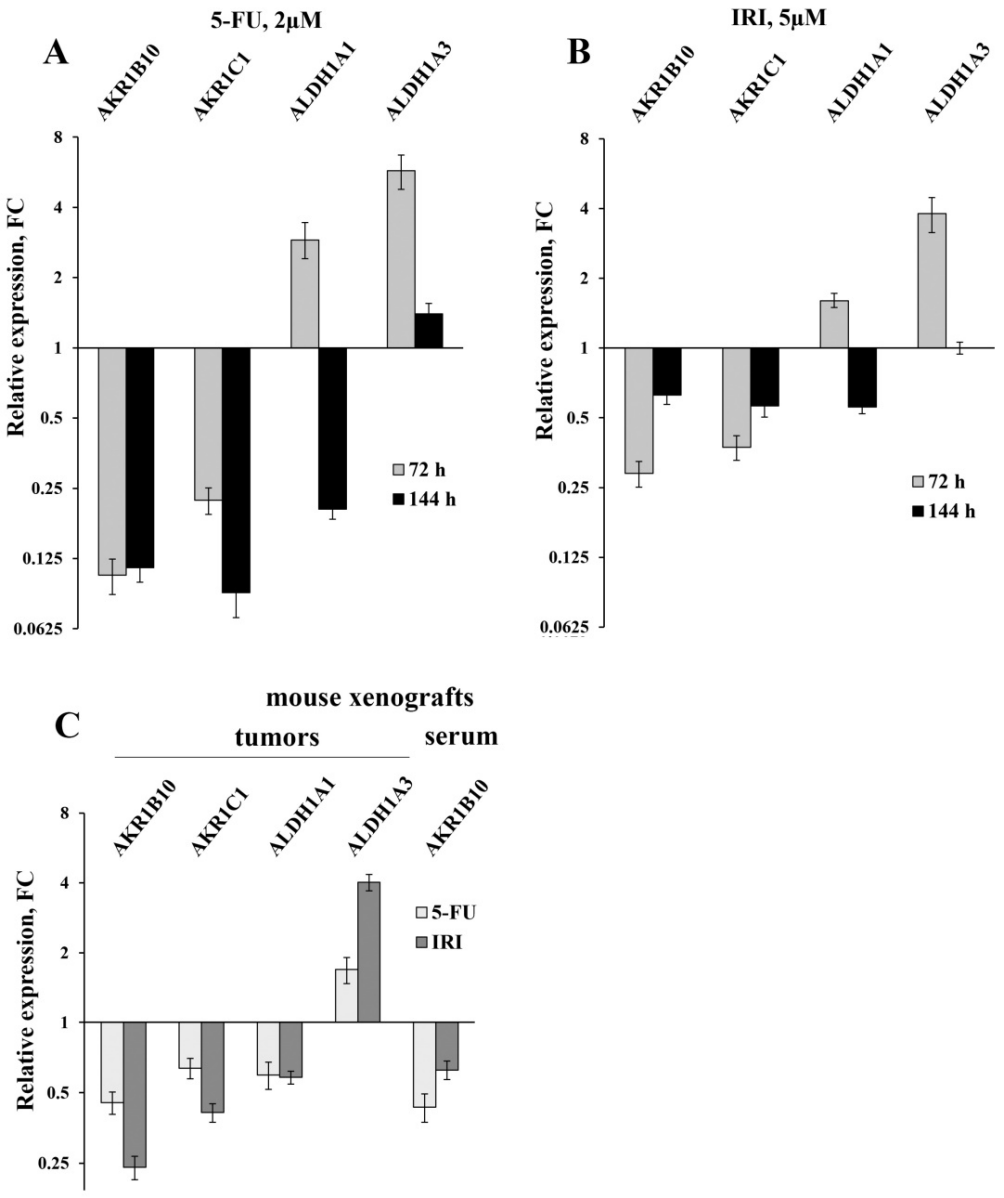
The results of the RT-qPCR of four selected mRNAs in colon cancer cells and in tumors and sera of xenografted mice (error bars - standard deviation). HT-29 cells were treated with 2 µM 5-FU (**A**) and 5 µM IRI (**B**); **C** - the results of RT-qPCR of HT-29 xenografted tumors (first four bar pairs) and sera (the last pair) of mice treated with IRI and 5-FU (four mice were taken for each treatment and five control mice were injected with buffered saline).

**Figure 2:**
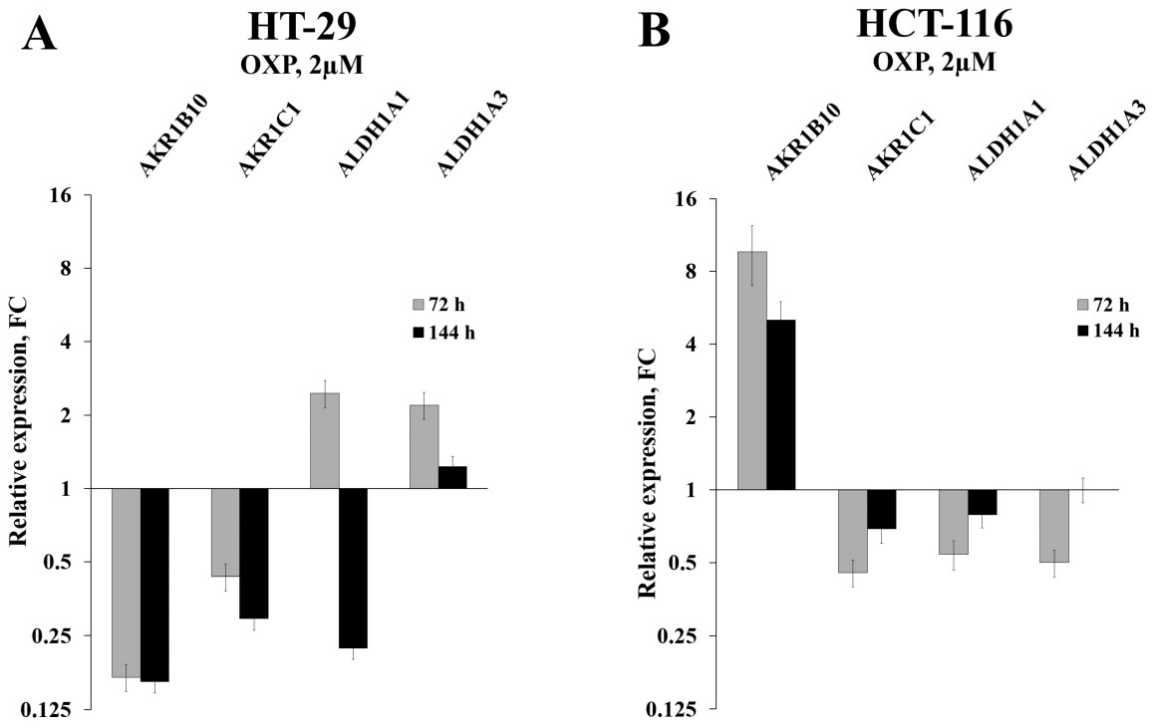
Relative expression levels of four selected mRNAs in HT-29 and HCT-116 cells treated with OXP for 72h, as detected by RT-qPCR (error bars - standard deviation).

Initially, we used RT-qPCR in order to measure the intracellular content of four key mRNAs that mainly determine the efficiency of detoxification of the ROS-generated aldehydes. We selected two ALDH-encoding mRNAs (ALDH1A1 and 1A3) and two AKR-encoding mRNAs (AKR1B10 and 1C1) and observed perfect coincidence of the RNA-Seq and RT-qPCR data. Next, we analyzed the expression levels of these mRNAs in HT-29 cells treated with three drugs (5-fluorouracil, oxaliplatin, and irinotecan), which are most frequently used for chemotherapy. We performed the analysis at different doses of the drugs and durations of treatment, both *in vitro* and *in vivo*. Obtained data demonstrated completely coincident transcriptional response of HT-29 cells treated with all three drugs *in vitro*: a significant reduction in the cellular content of AKR1B10 and AKR1C1 mRNAs (up to 10 times), an increased transcription of ALDH1A3 mRNA (up to five times), and multidirectional changes for ALDH1A1 mRNA (upregulation upon 72h treatment and downregulation at 144h treatment) (Figure 1A-B and Figure 2A). Next, using the same RT-qPCR format, we have compared transcriptional response of HT-29 (p53mut) and HCT-116 (p53wt) cells upon treatment with oxaliplatin at two concentrations and two treatment times. We have detected the strongest differences in the expression of *AKR1B10* gene, which occurred in the opposite directions in p53mut and p53wt cancer cells (five times down in HT-29 and ten times up in HCT-116) (Figure 2).

In order to provide *in vivo* validation of the drug treatment effect on expression of genes encoding four aldehyde-metabolizing enzymes we have treated mice carrying HT-29 tumor xenografts with 5-FU and IRI. In accordance with the results of cancer cell analysis, this experiment revealed diminished expression of AKR1C1 (FC=1.6-2.4) and ALDH1A1 mRNAs (FC=1.7), as well as upregulation of ALDH1A3 mRNA in xenograft tumors (FC=1.7-4.0) (Figure 1C). Importantly, we have observed a twofold decrease in the content of AKR1B10 mRNA in sera of 5-FU treated *vs* untreated animals (the remaining three mRNAs were undetectable).

### Bioinformatics analysis of transcriptional changes of the four selected mRNAs cancer cells treated with a wide spectrum of chemotherapeutics

**Figure 3:**
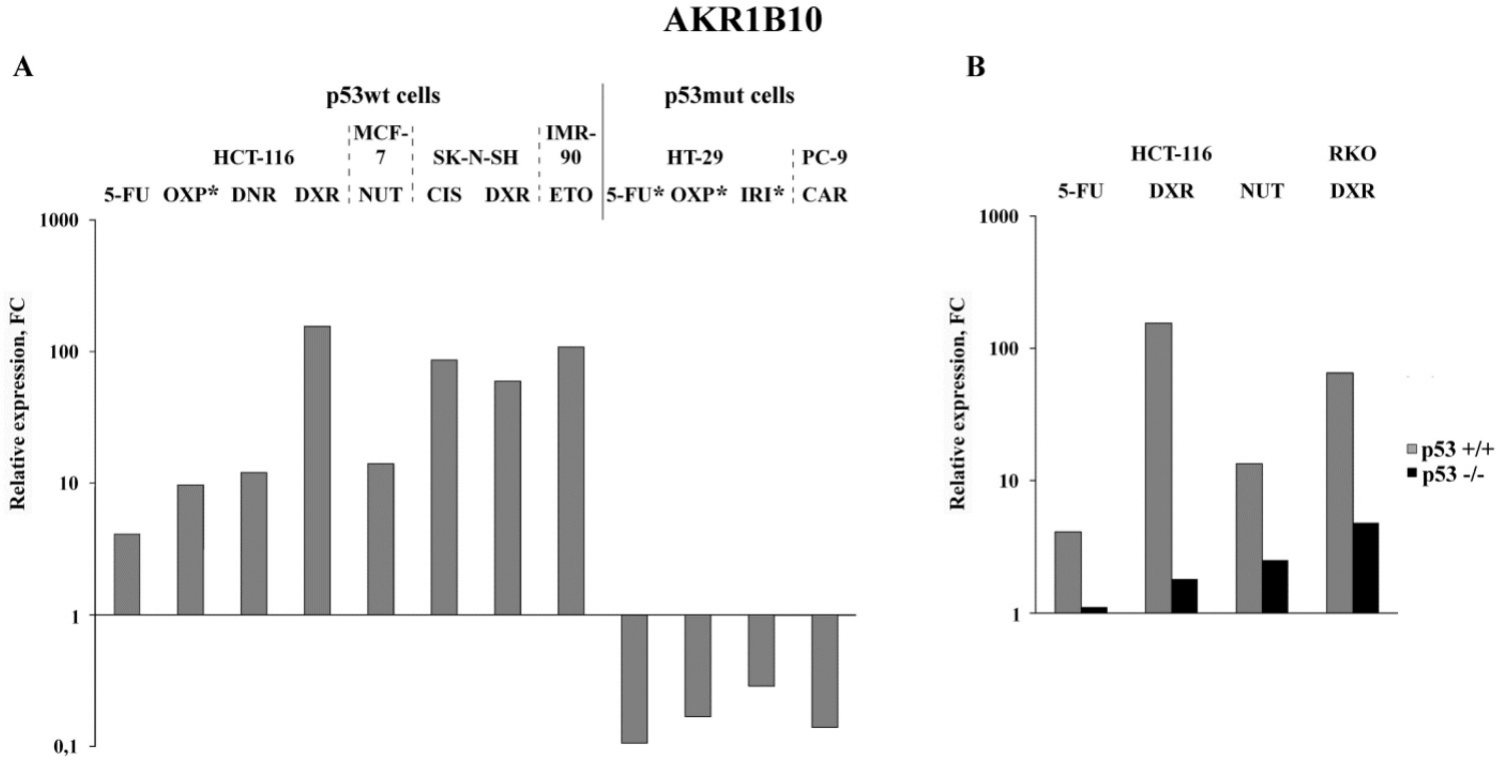
Bioinformatics analysis of public data deposited in NCBI Sequence Read Archive: expression alterations of *AKR1B10* in p53wt and p53mut cancer cell lines (**A**; * - results experimentally obtained in this study) and in paired p53wt cells and p53-knockout cells (**B**). Drugs: OXP, oxaliplatin; DNR, daunorubicin; DXR, doxororubicin; NUT, nutlin-3 (p53-activating imidazoline analog); CIS, cisplatin; ETO, etoposide; IRI, irinotecan; CAR, carboxyplatin. Cell lines: MCF-7, BRCA; SK-N-SH, neuroblastoma; PC-9, NSCLC; RKO, CRC; IMR-90, normal diploid fibroblasts.

Analysis of the published RNA-Seq data that were obtained upon treatment of various cancer cells with nine anticancer drugs demonstrated the highest (5 to 155-fold) increase in the AKR1B10 mRNA content in drug-treated p53wt cancer cells (Figure 3A). Importantly, its content dropped 2-8 times in cancer cells, which have gain of function mutation in the DNA binding domain of p53 (homozygous R273H mutation in HT-29 colon cancer cells or heterozygous R248Q mutation in PC-9 non-small lung cancer cells) (Figure 3A and Tables S3-S5). Similar transcriptional response (upregulation in p53+/+ cells and downregulation in p53 -/- cells) was observed by comparison of the RNA-seq data obtained upon drug treatment of paired p53wt CRC cell lines and their p53-knockouts (Figure 3B). The response of normal diploid IMR-90 fibroblasts to etoposide mimicked the response of p53wt cancer cells, which implies that changes in *AKR1B10* expression in response to anticancer drugs are dependent on their p53 status. Importantly, AKR1B10 mRNA content dropped significantly in cancer cells with gain of function mutation, as compared to p53wt cells. This implies that p53wt acts as an activator of AKR1B10 transcripion, whereas gain of function mutations in the p53mut lead to transcriptional repression of at least some of the p53 target genes.

Finally, we have compiled our experimental data with bioinformatics in order to compare the abundance of the four mRNAs encoding aldehyde-metabolizing enzymes in six untreated cancer lines of various origins and one line of normal diploid fibroblasts. Analysis of the average CPM values from the RNA-seq data and reflect mRNAs' content (Tables S2-S5) demonstrated that among the four selected genes the *ALDH1A3* gene is: 1) the only one that is robustly transcribed at moderate to high levels in five out of six analyzed cancer cell lines; 2) is by far the most highly expressed in normal diploid fibroblasts IMR-90 (Table 1).

### Consistent decrease in the AKR1B10 mRNA content in sera of colon cancer patients, as compared to sera of healthy donors

**Figure 4:**
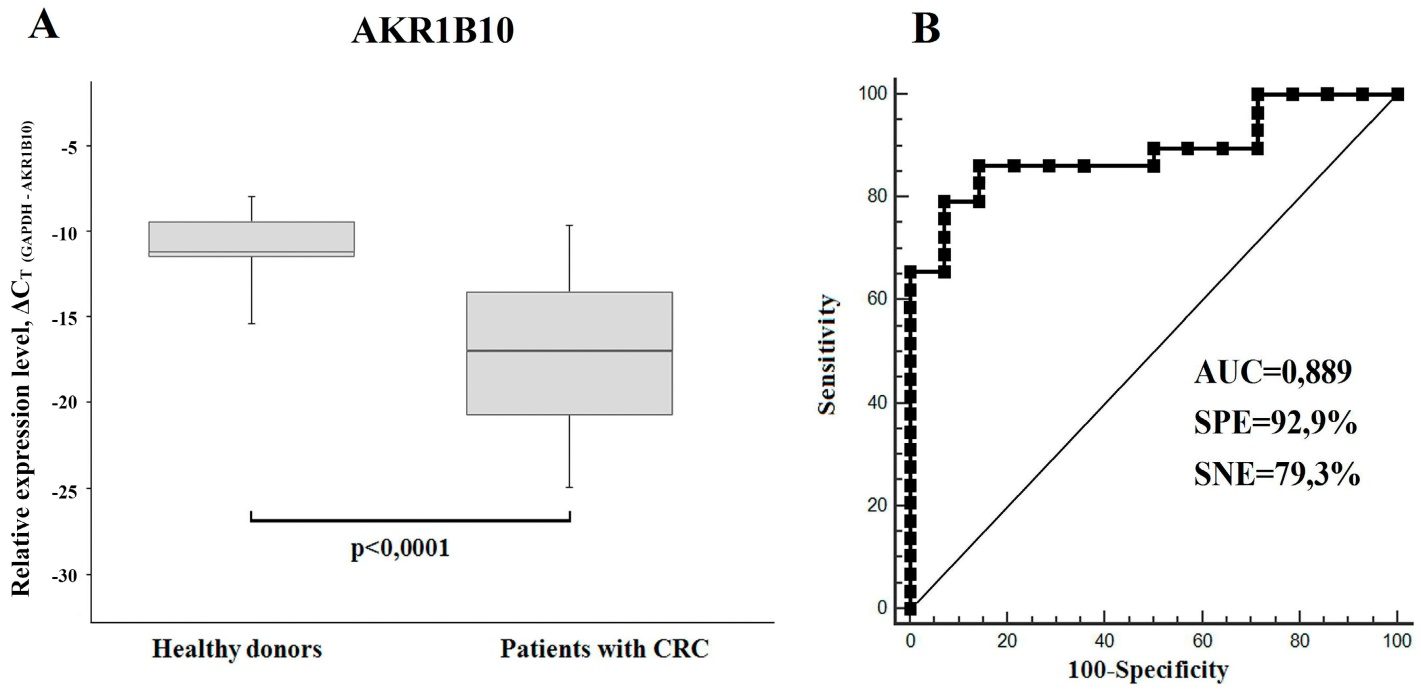
(A) AKR1B10 relative mRNA expression levels in sera of CRC patients (n=29) and healthy donors (*n* = 14) displayed as boxplots. GAPDH mRNA was used as control. Boxes indicate quartile range (25^th^-75^th^ percentiles), central line - median, whiskers - 10^th^ and 90^th^ percentiles. (B) Receiver operating characteristic curve (ROC) representing correlation between the amount of AKR1B10 mRNA in sera of CRC patients and healthy donors.

Using RT-PCR we have found a significant downregulation of AKR1B10 mRNA (FC=2.2-4.2) in total RNA samples isolated from sera of xenograft-bearing mice treated with either IRI or 5-FU (Figure 1C). Thereby, we compared the content of this mRNA in sera of CRC patients and healthy donors. In accordance with the xenograft experiments, we detected much lower levels of AKR1B10 mRNA in patients' sera, as compared to healthy donors (p<0.0001, FC=65.4). Calculated specificity (92.9%), sensitivity (79.3%), and AUC (area under the ROC curve; 0.889) were very high, which implies that AKR1B10 is a promising serological marker for CRC diagnosis (Figure 4).

## Discussion

Our data lead to several conclusions. First, we have found that transcription of *AKR1B10* undergoes the most significant changes upon oxaliplatin treatment of p53wt HCT-116 cells, as well as of p53mut HT-29 cells. However, the direction of the response was opposite: up in HCT-116 and down in HT-29. Bioinformatics search demonstrated that this phenomenon is also observed for most of other p53wt and p53mut cancer cells of various origins treated with a wide range of chemotherapeutic drugs. It is well established that p53 is a transcriptional activator of about 300 genes 23, including its *bona fide* target *AKR1B10* 24, which encodes one of the most effective enzymes catalyzing reduction of ROS-generated reactive aldehydes 2, 3. Notably, our data show that as opposed to the wild type p53, the mutant p53 protein, which contains missense gain of function mutation in the DNA binding domain (R273H in HT-29 or R248Q in PC-9 lung cancer cells), seems to act as a transcriptional repressor. This might explain the inhibition of *AKR1B10* transcription in p53mut CRC cell lines.

However, there is an alternative explanation of the opposite transcriptional response in drug-treated p53wt and p53mut cancer cells. Recently, it was found that only the mutant, but not the wild type p53 is capable of binding to the transcription factor NRF2 and that the p53mut-NRF2 complex can suppress transcription of most of the ARE-containing genes (including *AKR1B10* 25 by activation of proteasome gene transcription 26. Formation of the p53-NRF2 complex was shown to promote degradation of multiple tumor suppressor proteins (including p21, p27, the major effector of apoptosis NOXA, and the miRNA maturation factor KSRP). These data provide an alternative explanation for the opposite transcriptional response of the *AKR1B10* upon drug treatment of p53mut *vs*. p53wt cells, as well as explains the higher drug resistance of p53mut cells.

Second, though the quantity of mice was insufficient for generalized conclusion, we have detected a consistent *twofold* decrease in the AKR1B10 mRNA content in sera of four drug-treated xenograft-bearing mice, as compared to five untreated animals. Subsequent analysis of sera of healthy donors and colon cancer patients revealed a strong and reproducible decrease in the AKR1B10 mRNA abundance in patients' sera, which implies that this mRNA is a promising diagnostic marker of colorectal cancer. There are several previously described precedents, when mRNA or miRNA content was statistically lower in sera of cancer patients, as compared to sera of healthy donors 27, 28. One explanation of this unusual expression pattern consists in tumor-induced reprogramming of the leukocytes' transcriptome 27, another - in tumor exosome-directed reprogramming of the gastrointestinal epithelium cells, where *AKR1B10* is preferentially expressed. Further experiments will help to choose between these two alternatives.

Finally, we have found that among the four genes encoding aldehyde-metabolizing enzymes, only *ALDH1A3* is robustly expressed at moderate or high levels in most of the experimentally and bioinformatically analyzed cancer cell lines. Besides, this gene was shown to have the highest level of transcription in colonic tumors among all 19 genes of the ALDH family 29. It was shown that its knockdown in breast cancer cells MDA-MB-468 (containing a p53 gain of function mutation) does not alter cellular proliferation and drug resistance, but increases cell adhesion, migration, and ability to metastasize 30. Moreover, significant *ALDH1A3* upregulation was observed in HT-29 cells selected for the enhanced resistance to 5-FU. Selected cells were also found to have much higher resistance to many other ROS-producing chemotherapeutic drugs. However, the acquired drug resistance was inhibited by the *ALDH1A3* gene knockdown 29. Thereby, it seems that the ALDH1A3 protein is a key inactivator of ROS-generated aldehydes, whose activity determines drug resistance of cancer cells of various origins. This implies that this gene is a perspective target for the development of new chemotherapeutic drugs.

## Supplementary Material

Supplementary table 1.

Supplementary table 2.

Supplementary table 3.

Supplementary table 4.

Supplementary table 5.

## Figures and Tables

**Table 1:** Selected mRNAs' average content in untreated cells (NCBI SRA public data).

Cell line	mRNA CPM value (RNA-seq)			
	AKR1B10	AKR1C1	ALDH1A1	ALDH1A3
HT-29	80.0	10.1	827	18.1
PC-9	737	398	0.05	638
HCT-116	0.43	0.33	0.07	293
RKO	0.46	0	0	0.11
MCF-7	1.94	1.16	0	7.0
SK-N-SH	0.05	1.37	0.02	10.6
IMR-90	0.04	6.18	0.40	41.8

## Abbreviations

ALDH: aldehyde dehydrogenase
AKR: aldo-keto reductase
CRC: colorectal cancer
FC: fold change
FDR: false discovery rate
5-FU: 5-fluorouracil
IRI: irinotecan
OXP: oxaliplatin
ROS: reactive oxygen species
RT-qPCR: reverse transcription quantitative PCR
