# Supplementary material for: Treatment of cancer cells with chemotherapeutic drugs results in profound changes in expression of genes encoding aldehyde-metabolizing enzymes
Source: J Cancer. 2019 Jul 10;10(18):4256–63. doi: 10.7150/jca.32608 (PMC6691692; doi:10.7150/jca.32608)
Supplement: Supplementary file 1 — Supplementary table 1. [file jcav10p4256s1.pdf]

Table S1. Primer sequences for RT-qPCR

**Gene**	**F/R**	Primer sequence (5'- 3')	PCR product, bp	Genbank Access No.
1	2	3	4	5
AKR1B10	F	GGACCTGTTCATCGTCAGCAA	145	NM_020299.4
	R	CCCCAGACTTGAATCCCTGTG		
AKR1C1	F	GATGGCAGTGTGAAGAGAGAAGAC	121	NM_001353.5
	R	TCCTCACCTGGCTTTACAGACA		
ALDH1A1	F	AACTCCTCTCACTGCTCTCCACG	210	NM_000689.4
	R	GTCACCCTCTTCAGATTGCTTTTCC		
	R	CTTTAAGTAAGGACCGTGGCTCA		
ALDH1A3	F	TCAACTGCTACAACGCCCTCTAT	184	NM_000693.2
	R	CGCCGTCCGATGTTTGAG		
ACTB	F	CCTTCCTGGGCATGGAGTC	112	NM_001101.3
	R	CCAGACAGCACTGTGTTGGC		
GAPDH	F	GGAGTCAACGGATTTGGTC	181	NM_002046.3
	R	TGGGTGGAATCATATTGGAACAT		
